# Hydroxylumisterols, Photoproducts of Pre-Vitamin D3, Protect Human Keratinocytes against UVB-Induced Damage

**DOI:** 10.3390/ijms21249374

**Published:** 2020-12-09

**Authors:** Anyamanee Chaiprasongsuk, Zorica Janjetovic, Tae-Kang Kim, Cynthia J. Schwartz, Robert C. Tuckey, Edith K. Y. Tang, Chander Raman, Uraiwan Panich, Andrzej T. Slominski

**Affiliations:** 1 Department of Dermatology, University of Alabama at Birmingham, Birmingham, AL 35294, USA; anyamani.c@gmail.com (A.C.); zjanjetovic@uabmc.edu (Z.J.); tkim@uabmc.edu (T.-K.K.); schwartzc@acom.edu (C.J.S.); 2 Faculty of Medicine and Public Health, HRH Princess Chulabhorn College of Medical Science, Chulabhorn Royal Academy, Bangkok 10210, Thailand; 3 Department of Pharmacology, Faculty of Medicine Siriraj Hospital, Mahidol University, Bangkok 10700, Thailand; uraiwan.pan@mahidol.ac.th; 4 School of Molecular Sciences, The University of Western Australia, Crawley, WA 6009, Australia; robert.tuckey@uwa.edu.au (R.C.T.); edith.tang@uwa.edu.au (E.K.Y.T.); 5 Department of Medicine and Microbiology, Division of Clinical Immunology and Rheumatology, University of Alabama at Birmingham, Birmingham, AL 35294, USA; chanderraman@uabmc.edu; 6 VA Medical Center, Birmingham, AL 35294, USA

**Keywords:** photobiology, UV radiation, keratinocyte, inflammation, cell differentiation, lumisterol hydroxyderivatives

## Abstract

Lumisterol (L3) is a stereoisomer of 7-dehydrocholesterol and is produced through the photochemical transformation of 7-dehydrocholesteol induced by high doses of UVB. L3 is enzymatically hydroxylated by CYP11A1, producing 20(OH)L3, 22(OH)L3, 20,22(OH)2L3, and 24(OH)L3. Hydroxylumisterols function as reverse agonists of the retinoic acid-related orphan receptors α and γ (RORα/γ) and can interact with the non-genomic binding site of the vitamin D receptor (VDR). These intracellular receptors are mediators of photoprotection and anti-inflammatory activity. In this study, we show that L3-hydroxyderivatives significantly increase the expression of VDR at the mRNA and protein levels in keratinocytes, both non-irradiated and after UVB irradiation. L3-hydroxyderivatives also altered mRNA and protein levels for RORα/γ in non-irradiated cells, while the expression was significantly decreased in UVB-irradiated cells. In UVB-irradiated keratinocytes, L3-hydroxyderivatives inhibited nuclear translocation of NFκB p65 by enhancing levels of IκBα in the cytosol. This anti-inflammatory activity mediated by L3-hydroxyderivatives through suppression of NFκB signaling resulted in the inhibition of the expression of UVB-induced inflammatory cytokines, including IL-17, IFN-γ, and TNF-α. The L3-hydroxyderivatives promoted differentiation of UVB-irradiated keratinocytes as determined from upregulation of the expression at the mRNA of involucrin (IVL), filaggrine (FLG), and keratin 14 (KRT14), downregulation of transglutaminase 1 (TGM1), keratins including KRT1, and KRT10, and stimulation of ILV expression at the protein level. We conclude that CYP11A1-derived hydroxylumisterols are promising photoprotective agents capable of suppressing UVB-induced inflammatory responses and restoring epidermal function through targeting the VDR and RORs.

## 1. Introduction

Ultraviolet radiation (UVR) can mediate beneficial and harmful effects on the skin through a variety of biological responses [1,2]. UVB rays are responsible for the formation of vitamin D [3,4,5], however, UVB can also negatively affect the skin, inducing inflammation and interfering with cell differentiation [6,7]. During UVB exposure of the skin, the B ring of 7-dehydrocholesterol (7-DHC) is photolyzed, producing pre-vitamin D3 (pre-D3) with subsequent transformation to various photoisomers, including vitamin D3, tachysterol, and lumisterol (L3) [8,9,10]. Prolonged UVB exposure leads to the photochemical transformation of pre-D3 to L3, which involves the resealing of the B-ring, but in a different configuration to 7-DHC, making L3 a stereoisomer of 7-DHC [3,11].

It was assumed for many years that L3 was metabolically inactive, not affecting calcium metabolism nor having any significant biological activity. It was believed that the UVB-induced transformation of pre-D3 into L3 explained the lack of systemic intoxication by vitamin D3 produced in the skin with excessive sun exposure [3]. However, recently it has been demonstrated that L3 can be enzymatically hydroxylated to produce several biologically active products that are detectable in the human body [12,13,14]. One of the enzymes activating L3 is CYP11A1, best known for catalyzing the first step in steroid hormone synthesis. This enzyme is not only expressed in the classical steroidogenic tissues, such as the adrenal cortex, but at lower levels in several organs, including the skin and immune system [15,16,17,18]. CYP11A1 catalyzes the sequential hydroxylation of the side chain of L3, leading to the cutaneous production of several hydroxyl forms of L3, including 20(OH)L3, 22(OH)L3, 20,22(OH)_2_L3, and 24(OH)L3 [12,13]. Hydroxylumisterols have been reported to interact with the retinoic acid-related orphan receptors α and γ (RORα/γ), inhibiting RORγ- and RORα-mediated transcriptional activity [1,12]. The hydroxyderivatives of L3 are structurally very similar to other sterols and as well as acting as reverse agonists on RORα and RORγ, they interact with the non-genomic binding site of the vitamin D receptor (VDR) [12]. These receptors are all expressed in human skin [4,19,20,21].

VDR is a member of the family of nuclear receptors (NRs) [20,22,23]. NRs are ligand-activated transcription factors that regulate the expression of genes involved in physiological processes such as metabolism, reproduction, and inflammation [24]. Upon binding active forms of vitamin D3, the VDR heterodimerizes with the retinoid X receptor (RXR) [25]. This complex then binds to the vitamin D response element (VDRE) leading to activation of the transcription of vitamin D3-responsive target genes [26] involved in anti-inflammatory responses and cell differentiation [27]. Previous studies have demonstrated that RORs are implicated in several physiological and pathological processes in human organs, particularly the skin [28]. The first member of the ROR subfamily of NRs (RORα), named ‘retinoic acid receptor-related orphan receptor alpha’, has been identified as being similar to the retinoic acid receptor (RAR) and the retinoid X receptor (RXR) based on their sequence similarity [29]. RORα exhibits specific expression in skeletal muscle and skin [19], while RORγ is highly expressed in muscle tissue [30] and is reported to be a therapeutic target for inflammatory diseases [31,32]. In addition, the selective inhibition of RORs may be a promising therapeutic approach for the treatment of autoimmune diseases, metabolic disorders, and some cancers [33]. VDR/ROR pathways cause activation of IκB kinase β (IKKβ), which phosphorylates IκBα [34,35]. This causes dissociation of NFκB from the NFκB/IκB complex which enables NFκB to translocate into the nucleus and activate the expression of genes encoding proinflammatory cytokines, thus producing a variety of inflammatory responses [36]. The VDR and ROR can also stimulate keratinocyte differentiation with increased expression of biomarkers such as involucrin (IVL) and keratins [37].

Our previous research showed the effects of hydroxylumisterols on several pathways in different cells, as well as their ability to bind to RORα and RORγ receptors [12,13]. 20(OH)L3 was able to promote keratinocyte differentiation [12]. In addition, L3 and its hydroxyderivatives, including 20(OH)L3, 22(OH)L3, 20,22(OH)2L3, and 24(OH)L3, suppressed DNA damage and apoptosis signaling in epidermal keratinocytes [38]. However, the effects of these compounds on inflammatory responses and cell differentiation have not been elucidated yet. Therefore, we investigated the effects of hydroxylumisterols on the inflammatory responses and differentiation of keratinocytes exposed to UVB in the context of VDR and RORs expression.

## 2. Results

### 2.1. L3-Hydroxyderivatives Interact with the VDR and RORα and γ

**Figure 1:**
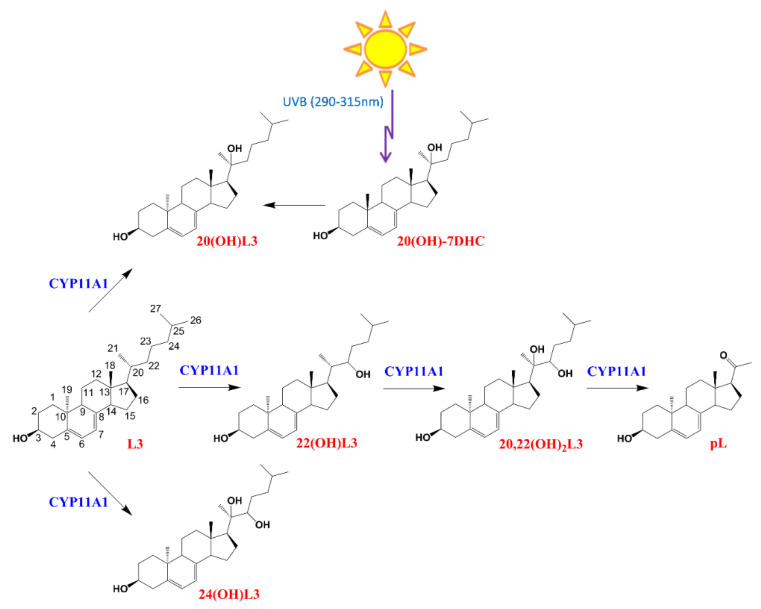
The chemical structures and pathway of synthesis of L3 hydroxyderivatives. pL, pregna-lumisterol.

CYP11A1 hydroxylates L3 at positions C-20, C-22, and C-24, with a small amount of cleavage of 20,22(OH)_2_L3 to produce pregna-lumisterol (Figure 1). 20(OH)L3 is also produced from 20(OH)7DHC through photochemical transformation after absorption of UVB by the B ring of the delta7 sterol (Figure 1). Previously, we documented that lumisterol hydroxyderivatives act as RORα and γ inverse agonists and interact with the non-genomic binding site of the VDR [12], indicating overlapping (action on RORs) and distinct (VDR) mechanisms of action compared with the corresponding vitamin D3 derivatives [1,19,39,40,41].

**Figure 2:**
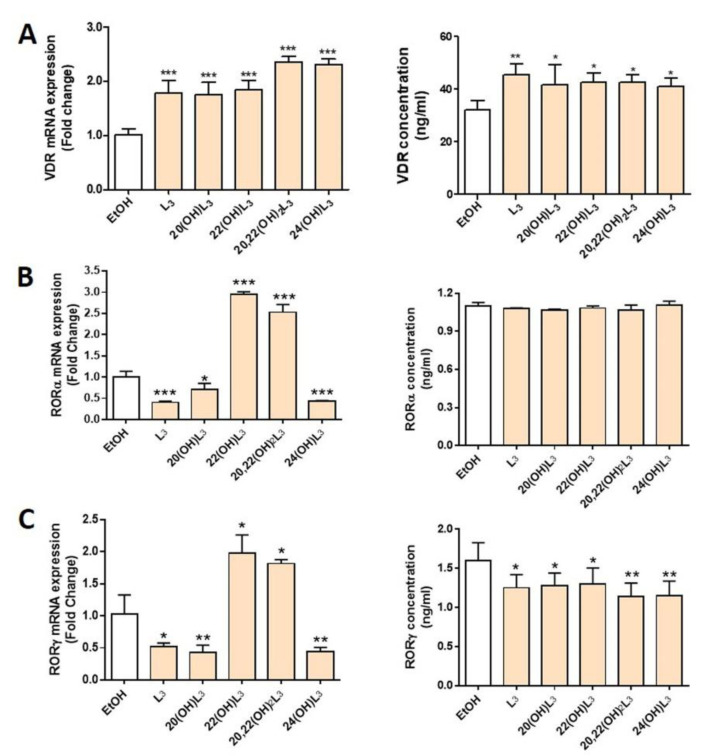
Modulation of the expression of mRNA for the vitamin D receptor (VDR) and retinoic acid-related orphan receptors α and γ (RORα/γ) in keratinocytes treated with L3 derivatives. Keratinocytes were treated with 100 nM lumisterols (shown as light orange bars) or ethanol (solvent control, shown as white bar) for 24 h. Real-time RT-PCR analysis and immunodetection were performed to measure (**A**) VDR, (**B**) RORα, and (**C**) RORγ mRNA and protein levels (ng/mL), respectively. The mRNA level of the targeted gene was normalized relative to the housekeeping genes, cyclopilin, and GAPDH mRNA levels. The concentrations of nuclear receptors (ng/mL) were measured by ELISA and were calculated from reading the absorbance at 450 nm from the appropriate standard curve. The statistical significance of differences was evaluated by the one-way ANOVA, * *p* < 0.05, ** *p* < 0.01, *** *p* < 0.001, for all conditions relative to the untreated ethanol control, *n* = 3.

In this study, we evaluated the expression of VDR and RORα/γ in human keratinocytes treated with L3 and its derivatives, including 20(OH)L3, 22(OH)L3, 20,22(OH)_2_L3, and 24(OH)L3. VDR mRNA and protein expression was significantly increased in all treated groups (Figure 2A). All L3 derivatives tested altered both mRNA and protein levels for RORγ, with all causing a significant decrease in RORγ protein levels (Figure 2C). However, for RORα, mRNA were altered, but not protein levels (Figure 2B). There were some variations in the effects of the different hydroxylumisterols, with the mRNA levels for RORα (RORA) and RORγ (RORC) being significantly reduced in cells treated with the L3, 20(OH)L3, and 24(OH)L3, but increased by 22(OH)L3 and 20,22(OH)_2_L3 (Figure 2B,C, left panel). This indicates that the site(s) of hydroxylation on the lumisterol side chain affects gene expression, and changes at the mRNA level are not always seen at the level of translation. Overall, these hydroxyderivatives stimulated the expression of VDR, while they inhibited the expression of RORγ.

### 2.2. The Effect of L3-Hydroxyderivatives on Nuclear Receptor Expression in Human Keratinocytes following UVB Irradiation

**Figure 3:**
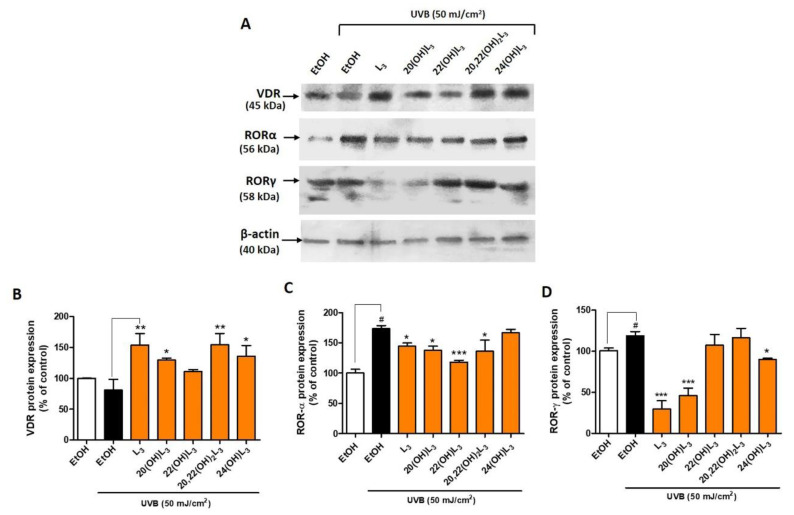
Changes in VDR and ROR protein expression in keratinocytes treated with L3 derivatives following UVB irradiation. Keratinocytes were pretreated with secosteroids (100 nM) or ethanol for 24 h before UVB irradiation (50 mJ/cm^2^) and then further incubated with the secosteroids for an additional 24 h. (**A**) Representative Western blots and (**B**–**D**) the densitometric analysis of protein expression shown as bar graphs. (**B**) VDR, detected at 45 kDa, (**C**) RORα, detected at 56 kDa, and (**D**) RORγ, detected at 58 kDa size by Western blotting. The protein levels were normalized relative to β-actin and presented as % of control and averaged from three-independent experiments (mean ± SD). The statistical analysis between the non-irradiated control (white bar) and UVB-irradiated cells (black bar) was calculated by Student’s *t*-test, and between UVB-irradiated control and lumisterol-treated cells (dark orange bars) by one-way ANOVA with Dunnett’s multiple comparison test. ^#^ *p* < 0.05 versus non-irradiated control cells and * *p* < 0.05, ** *p* < 0.01 and *** *p* < 0.001 versus irradiated and untreated cells.

Based on the evidence supporting a biological role of L3-hydroxyderivatives through binding to nuclear receptors, we further investigated their action on keratinocytes following UVB exposure. UVB is a key factor driving L3 production and may play a protective role against skin damage. The level of VDR protein was altered after the UVB irradiation (50 mJ/cm^2^) when compared to the non-irradiated cells (Figure 3). Conversely, treatment with L3, 20(OH)L3, 20,22(OH)_2_L3, or 24(OH)L3 significantly increased the expression of VDR protein (Figure 3A,B). 22(OH)L3 showed no effect on VDR levels. The expression of RORα and γ protein was significantly increased in UVB-irradiated cells compared to non-irradiated control cells (Figure 3). However, this effect was reversed when compared with ethanol-treated cells. RORα expression was significantly decreased in the L3-, 20(OH)L3-, 22(OH)L3-, and 20,22(OH)_2_L3-treated cells (Figure 3A,C). In addition, RORγ expression was significantly reduced in the L3-, 20(OH)L3-, and 24(OH)L3-treated cells compared to both untreated and treated cells (Figure 3A,D). As previously shown, RORs were upregulated while VDR was reduced in UVB-exposed cells [19,32,33,42]. These finding suggest that hydroxylumisterols can reverse the damaging effects of UVB on skin through upregulating VDR expression and downregulating RORα or RORγ expression. Activation of RORs, and RORγ in particular, leads to the pro-inflammatory phenotype [32], while activation of VDR has the opposite effect [43].

### 2.3. L3-Hydroxyderivatives Exert Anti-Inflammatory Activity in UVB-Irradiated Keratinocytes through NFκB/IκB-α Signaling

**Figure 4:**
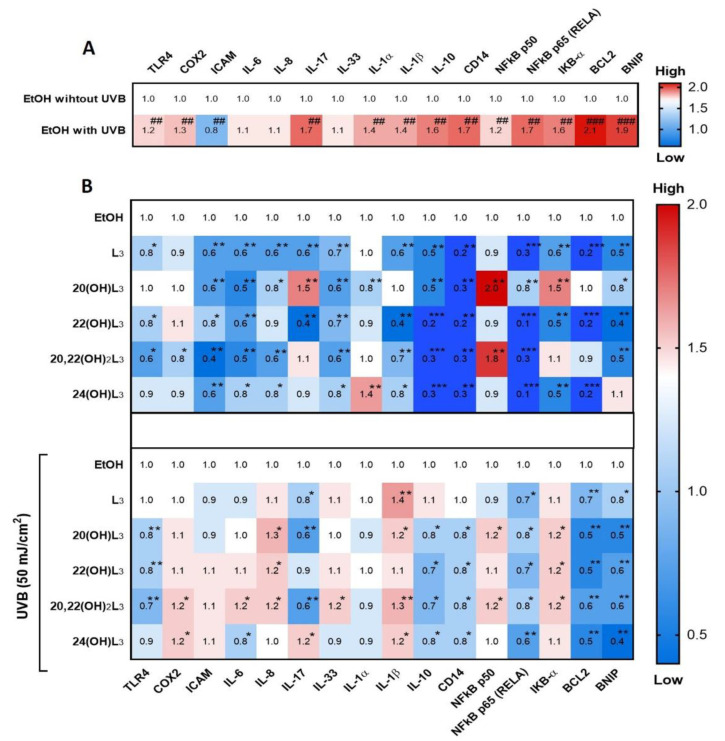
Heat map presentation of the changes in the expression of inflammatory genes by treatment of keratinocytes with L3 derivatives. Gene expression was normalized relative to β-actin, cyclophilin, and GAPDH mRNA. (**A**) Comparison of ethanol control groups between non-irradiated and irradiated groups. (**B**) Treatment groups compared to ethanol control group without or with UVB irradiation. Each vertical row represents the same gene product, and each horizontal row represents each sample. The fluorescence range, from high (red) to low (blue), is indicated by the colored bar and reflects the degree of fluorescence intensity/gene expression. Statistical analysis between the controls: non-irradiated and UVB-irradiated cells was done by Student’s *t*-test, and between cells treated with L3 derivatives and ethanol-only-treated control cells by one-way ANOVA with Dunnett’s multiple comparison test. ^##^ *p* < 0.01, ^###^ *p* < 0.001 versus non-irradiated control cells, and * *p* < 0.05, ** *p* < 0.01, and *** *p* < 0.001 versus irradiated ethanol-treated control cells, *n* = 3.

Skin inflammation is one of the basic characteristics of skin damage caused by UV. The RORs have been reported to be a mediator of UVB-induced inflammatory responses of the skin and are upregulated in keratinocytes, as well as in mouse skin exposed to UVB [44]. Hence, we studied the photoprotective effects of hydroxylumisterols on UVB-induced damage to keratinocytes through their anti-inflammatory activities. We investigated the dynamic fold changes in expression of sixteen genes involved in inflammatory responses. UVB irradiation (50 mJ/cm^2^) significantly induced expression of the genes encoding TLR4, COX2, IL17 (IL-17), IL1A (IL-1α), IL1A (IL-1β), IL10 (IL-10), CD14, NFKB1 (NFkB-p50), RELA (NFkB-p65), IKBA (IkB-α), BCL2, and BNIP (Figure 4A). The expression of eleven of the sixteen genes was strongly inhibited by treatment with hydroxylumisterols in non-irradiated cells: these include RELA (NFkB p65) and several interleukin genes (IL) (Figure 4B, upper map). Most of the lumisterol compounds tested also inhibited the expression of inflammatory genes following UVB exposure (Figure 4B, lower map). RELA expression was significantly decreased under all treatments in the UVB-irradiated cells (Figure 4B, lower map), suggesting that anti-inflammatory activities of all the lumisterol derivatives involved modulation of the NFκB p65.

**Figure 5:**
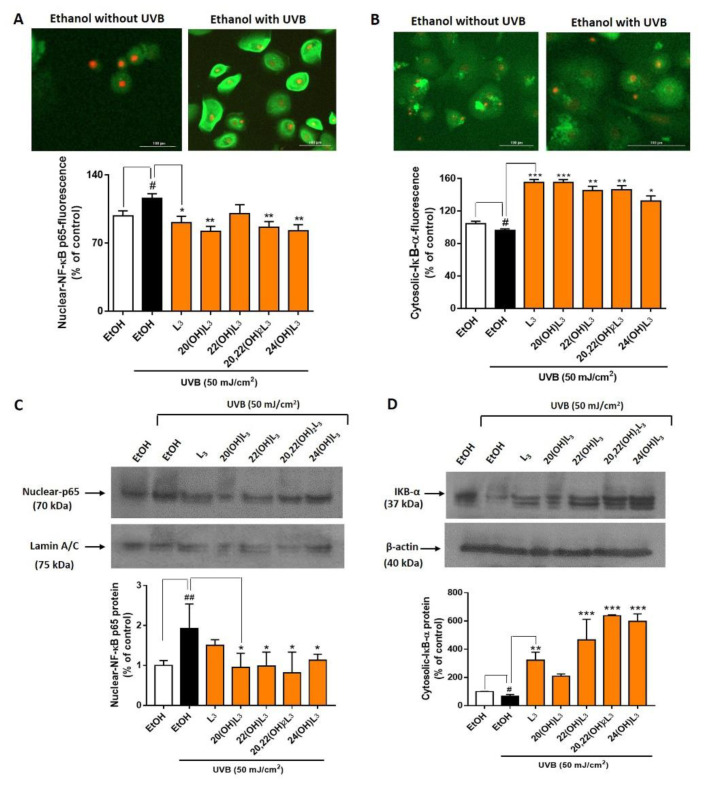
Lumisterol derivatives decrease nuclear NF-κB p65 levels and increase cytosolic levels of IκB-α in keratinocytes irradiated with UVB. Fluorescent microscopy of cells stained with (**A**) NF-κB p65 and (**B**) IκB-α antibodies were detected by the Cytation™ 5 cell imaging, *n* ≥ 100 cells for each condition. Scale bar = 100 µm. The single-color staining represents the expression in cytosol (green) and in nuclei (red), whereas the dual expression (orange–yellow) represents the expression in both cytosol and nucleus. Data are presented as the % of control for the nuclear-NF-κB p65 and the cytosolic-IκB-α (mean ± SD). (**C**) Western blot analysis of nuclear-NF-κB p65 (70 kDa) and (**D**) cytosolic-IκB-α (37 kDa). Data were normalized with lamin A/C (nuclear protein) and β-actin (cytosolic protein) and are presented as % of control for nuclear-NF-κB p65 or cytosolic-IκB-α expression (mean ± SD, *n* = 3). The statistical significance of differences between the non-irradiated control (white bar) and UVB-irradiated cells (black bar) was evaluated by Student’s *t*-test, and between UVB-irradiated controls and cells treated with lumisterols (dark orange bars) by one-way ANOVA with Dunnett’s multiple comparison test. ^#^ *p* < 0.05, ^##^ *p* < 0.01 versus non-irradiated control cells. * *p* < 0.05, ** *p* < 0.01, and *** *p* < 0.001 versus untreated cells exposed to UVB.

There are five members of the NFκB (Nuclear Factor κB) transcription factor family: Rel (c-Rel), RELA (p65), RelB, NFκB1 (p50 and its precursor p105), and NFκB2 (p52 and its precursor p100). NFκB dimers and heterodimers containing p65 function as activators for the transcription of NFκB family proteins [45,46]. At the onset of inflammation, NFκB p65 is associated with the inhibitor of kappa B (IκB) protein. IκB binds NFκB p65 and retains it in the cytoplasm by forming an inactive complex. Inflammatory signals activate I kappa kinase (IKK) to phosphorylate IκB [47]. This leads to the release of IκB from NFκB p65 and its degradation through the ubiquitin pathway. The released NFκB p65 translocates into the nucleus to activate the transcriptional process. Thus, degradation of IκB is a key early step for activation of inflammatory responses. 20(OH)L3, 22(OH)L3, and 20,22(OH)_2_L3 induced expression of IKBA (IκBα) mRNA following UVB irradiation (Figure 4B, lower map). We therefore evaluated changes in the localization and expression of NFκB p65/IκBα components. UVB-irradiation induced a significant increase in levels of nuclear-NFκB p65 (Figure 5A) and this was associated with a decrease in cytosolic-IκBα proteins compared to non-irradiated cells (Figure 5B). Treatment with L3, 20(OH)L3, 20,22(OH)_2_L3, and 24(OH)L3 significantly inhibited the UVB-induced translocation of NFkB p65 into the nucleus, as determined by immunofluorescence (Figure 5A, bar graph). This correlated with increased IκBα levels in the cytosol following treatment with L3 and its derivatives, including 20(OH)L3, 22(OH)L3, 20,22(OH)_2_L3, and 24(OH)L3 (Figure 5B, bar graph). These data were independently confirmed by Western blot analysis, showing that L3 and its hydroxyderivatives significantly reduced UVB-induced nuclear-NFκB p65 protein levels while simultaneously increasing IκBα protein in the cytosol, a mechanism that attenuates the inflammatory response (Figure 5C,D). All L3 compounds tested also markedly reduced translocation of NFkB p65 into the nucleus by increasing cytosolic-IkBα in non-irradiated cells (Supplementary Figure S1).

**Figure 6:**
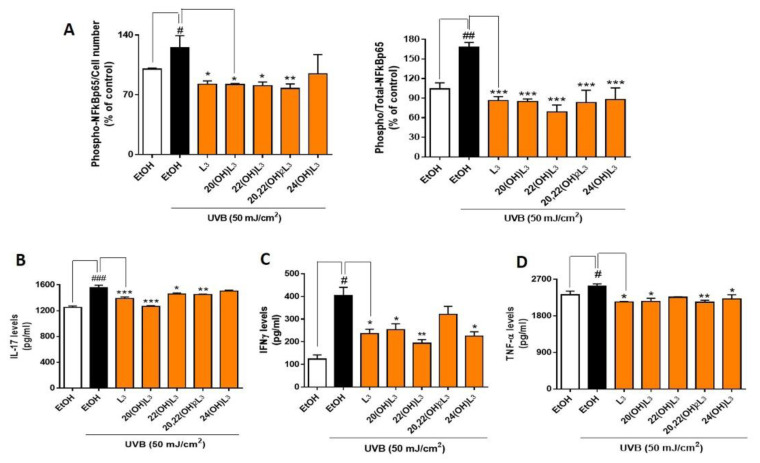
Hydroxylumisterols stimulate anti-inflammatory effects in UVB-irradiated keratinocytes by reducing NFκB p65 activity and production of pro-inflammatory cytokines. (**A**) ELISA using either phosphorylated-NF-κB p65 or total-NF-κB p65 antibody was used to measure the ratio of phospho-NF-κB p65/cell number and the ratio of phospho-NF-κB p65/total-NF-κB p65. The phosphorylation levels were then normalized against the ethanol control group and presented as the % of control (mean ± SD). ELISA assays using (**B**) IL-17, (**C**) IFNγ, or (**D**) TNF-α antibody were performed to evaluate the levels of inflammatory cytokines (pg/mL; mean ± SD) following treatments. The statistical analysis between the controls: non-irradiated cells (white bar) and UVB-irradiated cells (black bar), was done by Student’s t-test, and between UVB-irradiated ethanol-treated and lumisterol-treated cells (dark orange bars) by one-way ANOVA with Dunnett’s multiple comparison test. ^#^ *p* < 0.05, ^##^ *p* < 0.01 and ^###^ *p* < 0.001 versus non-irradiated control cells. * *p* < 0.05, ** *p* < 0.01 and *** *p* < 0.001 ersus untreated cells exposed to UVB, *n* = 3.

Phosphorylation of NFκB p65 on Ser536 by protein kinase A (PKA) leads to its activation and translocation into the nucleus [48]. We therefore assessed if the photoprotective effects of hydroxylumisterols were inversely associated with phosphorylation of NFκB p65, leading to a decrease in the expression of inflammatory cytokines. To address this, we determined levels of phospho-NFκB p65 relative to the cell number and total NFκB p65, as well as the production of inflammatory cytokines by ELISA [49]. We found that the ratio of phospho-NFκB p65 to cell number and the ratio of phospho-NFκB p65 to total-NFκB p65 were significantly increased in the UVB-irradiated group compared to non-irradiated control cells (Figure 6A). L3 and its hydroxyderivatives, including 20(OH)L3, 22(OH)L3, and 20,22(OH)_2_L3, inhibited the phosphorylation of NFκB p65 induced by UVB exposure (Figure 6A). Additionally, these hydroxylumisterols inhibited the phosphorylation of NFkB p65 in non-irradiated cells (Supplementary Figure S2). Almost all of the L3 compounds tested reduced the UVB-stimulated production of IL-17, IFN-γ, and TNF-α (Figure 6B–D). Overall, these findings show that hydroxylumisterols exert their anti-inflammatory effect by inhibition of NFκB p65 activation and consequently, production of inflammatory cytokines. The downregulation of IL-17 production may also be associated with inhibition of RORγ, which is a master regulator of IL-17 signaling [32].

### 2.4. L3-Hydroxyderivatives Promote Differentiation of UVB-Irradiated Keratinocytes

**Figure 7:**
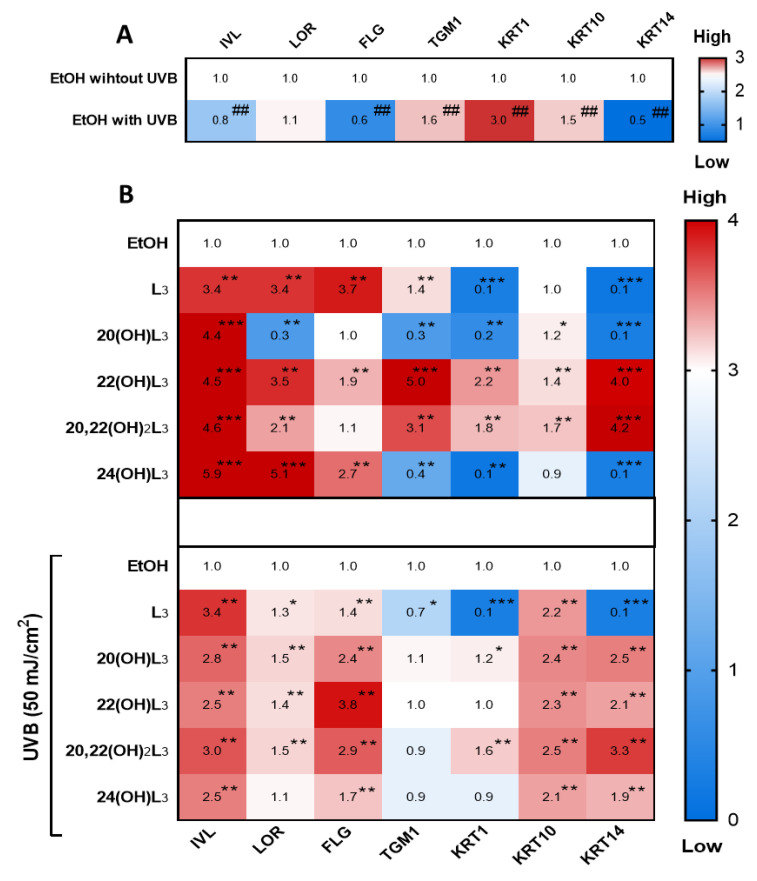
Heat map presentation of the changes in the expression of genes indicative of keratinocyte differentiation. Gene expression was normalized relative to β-actin, cyclophilin, and GAPDH mRNA. (**A**) Comparison of ethanol-treated control cells with non-irradiated and irradiated cells. (**B**) Treatment groups compared to ethanol-treated control cells without or with UVB irradiation. Each vertical row represents expression of the same gene product, and each horizontal row represents each sample tested. The fluorescence range, from high (red) to low (blue), is indicated by the colored bar and reflects the degree of fluorescence intensity/gene expression. The statistical analysis between the non-irradiated control and UVB-irradiated cells was done by Student’s t-test, and between lumisterol-treated cells and ethanol control cells by one-way ANOVA with Dunnett’s multiple comparison test. ^##^ *p* < 0.01 versus non-irradiated control cells, and *p < 0.05, ** *p* < 0.01, and *** *p* < 0.001 versus ethanol control cells, *n* = 3.

The repair of damaged skin requires cell differentiation to maintain cell turnover/regeneration. Key genes involved in the differentiation processes in keratinocytes are IVL, LOR, FLG, TGM1, KRT1, KRT10, and KRT14 [12,50]. We therefore evaluated the effects of the L3 compounds on the cell differentiation program in keratinocytes (Figure 7). The hydroxylumisterols strongly stimulated IVL mRNA expression in non-irradiated cells (Figure 7B, upper map). For UVB-irradiated cells, there was a significant modulation of the expression of most of the genes analyzed: an upregulation of IVL, FLG, and KRT14, and a downregulation of TGM1, KRT1, and KRT10 (Figure 7A). Following treatment, all derivatives significantly stimulated IVL, FLG, and KRT10 mRNA expression in the UVB-irradiated cells (Figure 7B, lower map).

**Figure 8:**
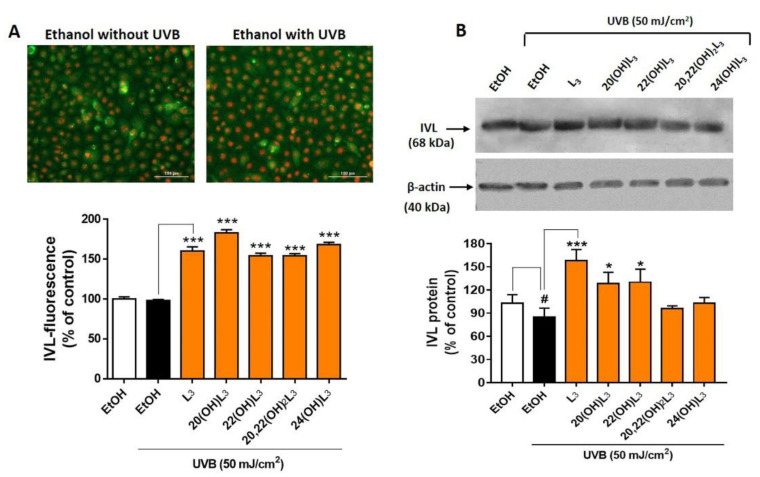
Lumisterol hydroxyderivatives stimulate the differentiation in UVB-irradiated keratinocytes, as shown by increased involucrin (IVL) protein expression. (**A**) Fluorescent microscopy of cells stained with IVL antibodies was detected by the Cytation™ 5 cell imaging, *n* ≥ 100 cells for each condition. Scale bar = 100 µm. The single-color staining represents the expression in cytosol (green) and in nuclei (red), whereas the dual expression (orange–yellow) represents the expression in both cytosol and nucleus. (**B**) Western blot analysis of IVL (68 kDa). Data were normalized relative to β-actin (loading control) and are presented as the % of ethanol control (mean ± SD, *n* = 3). The statistical significance of differences between the control non-irradiated cells (white bar) and the UVB-irradiated cells (black bar) was evaluated by Student’s t-test, and between UVB-irradiated control and lumisterol-treated cells (dark orange bars in the chart) by one-way ANOVA with Dunnett’s multiple comparison test. ^#^ *p* < 0.05 versus non-irradiated control cells. * *p* < 0.05 and *** *p* < 0.001 versus untreated cells exposed to UVB.

Based on the drastic upregulation of the expression of IVL shown by the heat map analysis (Figure 7), expression of this biomarker at the protein level was measured to confirm the biochemical effects of the hydroxylumisterols. All compounds tested promoted keratinocyte differentiation in the UVB-irradiated cells compared to untreated cells (Figure 8A, bar graph). Western blot analysis of IVL protein showed a reduction in keratinocyte differentiation following UVB irradiation when compared to non-irradiation cells (Figure 8B). As for the immunofluorescence of IVL, the IVL protein levels were significantly increased by all the lumisterol compounds tested (Figure 8B). All lumisterols also significantly promoted keratinocyte differentiation without UVB exposure, as shown by the immunofluorescence analysis of involucrin, a hallmark of differentiation (Supplementary Figure S3). Thus, hydroxylumisterols promote keratinocyte differentiation, as seen by the upregulation of IVL levels in both the presence and absence of UVB irradiation.

## 3. Discussion and Conclusions

Our previous investigations on the effects of lumisterol (L3) on nuclear receptors showed that its hydroxyderivatives could serve as partial agonists on the non-genomic site of the VDR and as inverse agonists on the RORα and RORγ [1,12]. In addition, the role of the VDR and RORs in inflamed and damaged skin have been studied. Hence, the photoprotective pathways of L3 and its hydroxyderivatives acting via VDR and RORα/γ might be potential targets for the development of therapeutic agents for skin disorders associated with the inflammation and impaired differentiation of keratinocytes. In this study, we demonstrated that all the lumisterol compounds tested promote the expression of the VDR, whereas some derivatives inhibited expression of the RORs following UVB exposure. Some variation was observed between the actions of the lumisterol compounds on the downregulation of the RORs in keratinocytes exposed to the UVB irradiation. For example, treatment with L3, 20(OH)L3, 22(OH)L3, and 20,22(OH)_2_L3 specifically reduced the expression of RORα protein, whereas the treatment with L3, 20(OH)L3, and 24(OH)L3 decreased RORγ protein expression in the UVB-irradiated cells. Thus, 22(OH)L3 and 20,22(OH)_2_L3 reduce the expression of RORα, 24(OH)L3 reduces RORγ, while L3 and 20(OH)L3 reduce the expression of both RORα and RORγ.

UVB is one of the most important external stimuli promoting skin damage via several pathways, including the inflammatory response. Exposure to high UVB radiation could result in the activation of transmembrane proteins such as toll-like receptor 4 (TLR4) and cluster of differentiation 14 (CD14), subsequently promoting the phosphorylation of IκB, promoting NFκB release from the NFκB/IκB complex. Subsequent translocation of NFκB into the nucleus induces the expression of genes encoding inflammatory response proteins, e.g., cytokines, chemokines, intercellular adhesion molecule (ICAM), and cyclooxygenase-2 (COX-2) [36,51]. The NFκB transcriptional activity promotes the secretion of inflammatory cytokines and chemokines outside the cells, such as a variety of interleukin (IL) subtypes including IL-6, IL-8, IL-17, IL-33, IL-1α, IL-1β, IL-10, IFNγ, and TNF-α [52]. During the inflammatory response, apoptosis can be induced through the B-cell lymphoma 2/adenovirus E1B-19K protein-interacting protein (Bcl2/BNIP) signaling pathway [53]. Since the VDR and RORs are involved in modifying inflammatory responses in damaged skin, we evaluated the biomarkers described above to test the anti-inflammatory properties of the hydroxylumisterols in keratinocytes exposed to UVB. The results indicated that the lumisterols exhibit photoprotective effects on UVB-irradiated keratinocytes by inhibiting NFκB p65 expression and increasing the expression of IκBα. The result is a reduction in the levels of IL-17, IFN-γ, and TNF-α inflammatory cytokines.

Keratinocyte differentiation plays an important role in skin repair and regeneration. Therefore, this represents a target for protective effects against skin damage. Involucrin (IVL) is a marker of the terminal differentiation of keratinocytes [54]. A previous study showed that inflammatory cytokines have the ability to decrease cytokeratin 10 in keratinocytes [55]. In addition, our previous study demonstrated the upregulation of IVL and KRT10 mRNA levels in non-irradiated keratinocytes treated with the 20(OH)L3 [12]. In this study, we found that the hydroxylumisterols promote expression of differentiation biomarkers, including involucrin, in keratinocytes irradiated with UVB. This indicates that one of the mechanisms by which these compounds provide photoprotection is by promoting skin regeneration. Phytochemicals have similarly been used for skin protection since they act as photoprotective agents by modulating cellular defense systems via their antioxidant and anti-inflammatory properties [56,57,58]. Our findings on the photoprotective properties of CYP11A1-derived hydroxylumisterols are consistent with data from Mason and coworkers, who showed that the chemically synthesized lumisterol derivative 1,25(OH)_2_L3, like 1,25(OH)_2_D3, shows protective properties against UVR [59,60,61,62].

In conclusion, the CYP11A1-derived hydroxylumisterols exert photoprotective effects on keratinocytes, suppressing the inflammatory response and stimulating keratinocyte differentiation via targeting VDR and ROR nuclear receptors. To further evaluate the pharmacological properties of these hydroxylumisterols for use as photoprotective and anti-inflammatory agents, future studies using in vivo models are warranted.

## 4. Materials and Methods

### 4.1. Source of Hydroxylumisterols

Lumisterol (L3) was purchased from Toronto Research Chemicals (North York, ON, Canada). 22(OH)L3, 20,22(OH)_2_L3, and 24(OH)L3 were enzymatically synthesized from the action of CYP11A1 on L3 as described previously [13]. While this procedure produces some 20(OH)L3, this sterol was also photochemically synthesized as described previously [63,64]. The scheme for enzymatic/chemical synthesis and the chemical structures of the hydroxylumisterols used in this study are shown in Figure 1.

### 4.2. Cell Culture and Treatment

Human epidermal keratinocytes (HEKn) isolated from neonatal foreskin of African American donors were cultured as previously described [45,65]. HEKn were grown on the collagen-coated plates in the presence of the EpiGROTM human epidermal keratinocyte media supplemented with EpiGROTM human epidermal keratinocyte supplements (Millipore, Burlington, MA, USA). Test compounds, including L3, 20(OH)L3, 22(OH)L3, 20,22(OH)_2_L3, or 24(OH)L3, were dissolved in ethanol prior to the treatment. HEKn were then treated with 0.1% ethanol (vehicle control group) or 100 nM of each test compound (1:1000 dilution of the stock concentration in the keratinocyte media containing 0.5% bovine serum albumin or BSA) for 24 h before UVB irradiation. Media were then replaced with phosphate buffered saline (PBS) prior to the UVB exposure (UV transilluminator 2000 from Bio-Rad, Hercules, CA, USA) at a dose of 50 mJ/cm^2^, as previously described [38,66]. After UVB irradiation, cells were incubated in fresh medium supplemented with the above hydroxylumisterols for an additional 24 h.

### 4.3. Real-Time RT-PCR and Heat Map Analysis of mRNA Expression

The HEKn cells were treated with the ethanol vehicle (dilution 1:1000) or hydroxylumisterols (100 nM) for 24 h prior to the UVB irradiation. After washing with PBS, the cells were then irradiated with 50 mJ/cm^2^ of UVB and further incubated in fresh medium supplemented with the same derivatives for an additional 24 h. Cell pellets were used to isolate the RNA using the absolutely RNA RT-PCR Miniprep kit (Stratagene, La Jolla, CA, USA). All real-time RT-PCR reactions were performed in triplicate using the Kapa SYBR Fast qPCR Master Mix (Kapa Biosystems, Boston, MA, USA) containing adequate primers. The sequences of the PCR primer sets for each gene are shown in Supplementary Table S1. The mean of fold-change of the expression of each gene was normalized relative to the three housekeeping genes separately (β-actin, cyclopilin, or GAPDH mRNA) utilizing the ΔΔCt method. Changes in gene expression are presented as the mean of the fold-change with statistically significant differences indicated. The Student’s t-test was used for the comparison between the non-irradiated or irradiated control cells. The *p*-value is reported as * *p* < 0.05, ** *p* < 0.01, *** *p* < 0.001, and **** *p* < 0.0001. The heat map of each gene was graded and generated using Prism5 (GraphPad Software Inc., San Diego, CA, USA).

### 4.4. Phospho-NFkB p65 Detection by the InstantOne ELISA

The pellets of the treated cells (as described above) were collected to measure NFκB phosphorylation by using the NFκB p65 (Total/Phospho) Human InstantOne™ ELISA Kit (Cat No. 85-86083-11, Thermo Fisher Scientific, Waltham, MA, USA). An equal volume of the capture antibody reagent and the detection antibody reagent was prepared prior to the experiment. In each well, 50 μL of whole cell lysate, a cell lysis mix (negative control), and a positive control cell lysate were individually added onto the pre-coated ELISA plate. Two types of capture antibody reagents, total-NFκB p65 antibody and phospho-NFκB p65 antibody, were separately incubated in each well of the pre-coated plate containing sample lysates (50 μL/well) for 1 h. The plate was washed before adding a detection reagent for 10–30 min and the detection reaction was sequentially inhibited by adding a stop solution (100 μL/well). The absorbance was immediately measured at 450 nm. The phospho-NFκB levels and the ratio of phospho-NFκB p65 vs. total-NFκB p65, normalized with the cell numbers, were then calculated. Data were compared with the control groups (ethanol without UVB or ethanol with UVB) and presented as the percentage (mean ± standard deviation (SD)) of control.

### 4.5. Immunofluorescent Analysis of NFκB p65, IκB-α, and IVL Expression

The treated cells were fixed in 4% paraformaldehyde (PFA) and incubated in blocking solution (1% BSA in PBS) for 1 h at room temperature. The cells were then incubated with the primary antibody (as indicated in Supplementary Table S2) at a dilution of 1:200 in blocking solution overnight at 4 °C. Alexa-Fluor 488 fluorescence secondary antibody solution (Supplementary Table S2) was then added, and samples were incubated for 1 h. After washing with PBS, the nuclei were stained red with propidium iodide (PI) (Vector Laboratories, Burlingame, CA, USA). The stained cells were imaged at 40X magnification and analyzed with Gen5.0 software with a Cytation™ 5 cell imaging multi-mode reader. Fluorescence intensity was analyzed using ImageJ software by the measurement of quantitative data in a green fluorescence channel. The quantitative data from ImageJ was used to calculate the percentage (mean ± SD) of control (ethanol without UVB or ethanol with UVB).

For the analysis of nuclear protein expression, the fluorescent intensity of the cell nuclei was measured separately from the fluorescence of the whole cells. Fluorescent staining in the nuclear fractions was measured as the PI-stained cells, while the fluorescent staining in the cytosolic fractions was calculated by subtracting the nuclear staining from the whole cell staining. The fluorescent intensities were calculated as previously described [67,68]. Data were normalized using the protein levels of control groups (ethanol without UVB or ethanol with UVB) and presented as the percentage (mean ± SD) of control.

### 4.6. Nuclear Receptor Protein Expression and the Measurement of Pro-Inflammatory Cytokines

Following treatment, cell supernatants were collected to measure the protein levels of the nuclear receptors, including VDR, RORα, and RORγ, by using ELISA assay kits (catalog number MBS266752, MBS4503748, and MBS4502279, respectively (MyBioSource, Inc., San Diego, CA, USA)). The cell supernatants were collected to measure the cytokines, including IL-17, IFN-γ, and TNF-α levels, by using ELISA assay kits (catalog number RAB0262, RAB022, and RAB1089, respectively (Sigma-Aldrich, St. Louis, MO, USA)). Standard solution, or cell samples comprising the cell extracts or the cell supernatants (100 µL), was added to each type of pre-coated 96-well plate and incubated overnight at 4 °C. The plates were then incubated with each of the detection antibodies: VDR, RORα, RORγ, IL-17, IFN-γ, and TNF-α (100 µL/well) for 1 h at room temperature. Streptavidin solution (100 µL) was then added to each well and plates were incubated for 45 min. After the antibody-HPR incubation, TMB one-step substrate reagent (100 µL) was added to the wells and plates were further incubated for 30 min before the addition of a stop solution (50 µL/well). The absorbance was immediately measured at 450 nm. The absorbance value is proportional to the protein levels of human VDR, RORs, or to the levels of the secreted cytokines in samples. Data were used for the calculation of the nuclear receptor levels (ng/mL) and the cytokines levels (pg/mL) by comparing the absorbance to an appropriate standard curve. Cytokine levels are presented as mean ± SD in histograms.

### 4.7. Western Blot Analysis of VDR/ROR Protein Levels and Measurement of Inflammation and Cell Differentiation Biomarkers

Cells were treated with ethanol vehicle (dilution 1:1000) or hydroxylumisterols (100 nM) for 24 h prior to UVB irradiation (50 mJ/cm^2^) and further incubated in fresh medium supplemented with the same derivatives for an additional 24 h. The treated cells were then washed with ice-cold PBS, mixed with phosphatase inhibitors, and removed from the dish by scraping. The cell pellets were collected by centrifuging at 2000 × *g* for 5 min, re-suspended in 200 µL of 1X hypotonic buffer (Active motif, Carlsbad, CA, USA), and further incubated on ice for 15 min. The lysate mixtures were centrifuged at 14,000× *g* for 30 s at 4 °C and the supernatant was collected as the cytosolic fraction. The pellets containing nuclei were then resuspended in 50 µL of the complete lysis buffer plus 2.5 µL of detergent for solubilizing membranes, and further incubated on ice with intermittent vortexing for 30 min. These samples were centrifuged at 14,000 × *g* for 10 min and the supernatant was collected as the nuclear fraction. The protein concentration of cytosolic and nuclear fractions was measured using the Bradford method (Bio-Rad, Hercules, CA, USA).

The proteins in extracts were separated by SDS-polyacrylamide gel electrophoresis using a Mini-PROTEAN^®^ TGX™ gel (Bio-Rad, Hercules, CA, USA) and transferred to a polyvinylidene fluoride (PVDF) membrane. Membranes were blocked with 5% skim milk and then incubated overnight at 4 °C with the primary antibodies, as shown in the list of antibodies used in the study (Supplementary Table S2). The membranes were then incubated with the appropriate HRP-conjugated secondary antibodies for 1 h at room temperature in TBST. Immuno-reactivity was detected using the Bio-Rad ECL kit Supersignal West Pico Chemiluminescent Substrate (Pierce). Protein expression was normalized relative to the loading controls. β-actin was used as an internal control for the whole cell extracts and cytosolic fraction, while Lamin A/C was used for the nuclear fractions. The antibodies used for the blots were then removed using Western blot stripping buffer (Thermo Fisher Scientific, Waltham, MA, USA) and membranes were re-probed with the other primary antibodies. Respective protein bands were identified from their size (kDa), as indicated in Supplementary Table S2. Band intensities were analyzed by ImageJ software (NIH free download) and normalized using the loading controls (lamin A/C protein for nuclear fractions and the β-actin for cytosolic and whole cell fractions), as well as using the protein levels of the control groups (ethanol without UVB or ethanol with UVB). Data are presented as the percentage (mean ± SD) of the control.

### 4.8. Statistical Analysis

Data are expressed as the mean ± SD of at least three separate experiments (*n* ≥ 3) performed on different days using freshly prepared reagents. The statistical significance between the controls, non-irradiated cells, and UVB-irradiated cells, was evaluated by the Student’s t-test, and between hydroxylumisterol-treated cells and ethanol-treated control cells by one-way analysis of variance (ANOVA) with Dunnett’s multiple comparison test using Prism (GraphPad Software Inc., San Diego, CA, USA). The *p*-value is reported as # *p* < 0.05, ## *p* < 0.01, and ### *p* < 0.001 versus non-irradiated control cells, and * *p* < 0.05, ** *p* < 0.01, and *** *p* < 0.001 versus ethanol-treated control cells.

## Supplementary Materials

The following are available online at https://www.mdpi.com/1422-0067/21/24/9374/s1.

## Abbreviations

L_3_	lumisterol_3_
20(OH)L_3_	20-hydroxylumisterol_3_
22(OH)L_3_	22-hydroxylumisterol_3_
20,22(OH)_2_L_3_	20,22-dihydroxylumisterol_3_
24(OH)L_3_	24-hydroxylumisterol_3_
NRs	nuclear receptors
VDR	vitamin D_3_ receptor
RORα	retinoic acid-related orphan receptor α
RORγ	retinoic acid-related orphan receptor γ
CYP11A1	cytochrome P450 family 11 subfamily A member 1
HEKn	human epidermal keratinocytes, neonatal
NF-κB	nuclear factor kappa B
IκB	inhibitor kappa B
TLR4	toll-like receptor 4
CD14	cluster of differentiation 14
COX-2	cyclooxygenase-2
IL	interleukin
IFNγ	interferon gamma
TNF-α	tumor necrosis factor-alpha
Bcl2	B-cell lymphoma 2
BNIP	B-cell lymphoma 2/adenovirus E1B-19K protein-interacting protein
IVL	involucrin
LOR	loricrine
FLG	filaggrine
TGM1	transglutaminase 1
*KRT1*	gene encoding cytokeratin 1
*KRT10*	gene encoding cytokeratin 10
*KRT14*	gene encoding cytokeratin 14
