# Expression of Concern: Chaiprasongsuk et al. Hydroxylumisterols, Photoproducts of Pre-Vitamin D3, Protect Human Keratinocytes Against UVB-Induced Damage. *Int. J. Mol. Sci.* 2020, *21*, 9374

**DOI:** 10.3390/ijms27083359

**Published:** 2026-04-09

**Authors:** IJMS Editorial Office

**Affiliations:** MDPI, St. Alban-Anlage 66, 4052 Basel, Switzerland; ijms@mdpi.com

With this notice, the *International Journal of Molecular Sciences* Editorial Office alerts the readers of concerns related to this article [1]. Following publication, concerns were raised to the Editorial Office regarding the potential image issues published in article [1], cited below.

The investigation is being coordinated by the Editorial Office, and the authors of this publication have been contacted for further clarification. The Editor-in-Chief has decided to issue this expression of concern while an investigation is being conducted by the authors’ institution, University of Alabama at Birmingham’s Office of Research.

Once this process is complete, the Editorial Office will provide an update on this situation and announce any post-publication modification deemed to be necessary, as per MDPI’s Updating Published Papers policy.
