# Supplementary material for: Hydroxylumisterols, Photoproducts of Pre-Vitamin D3, Protect Human Keratinocytes against UVB-Induced Damage
Source: Int J Mol Sci. 2020 Dec 9;21(24):9374. doi: 10.3390/ijms21249374 (PMC7763359; doi:10.3390/ijms21249374)
Supplement: Supplementary file 1 [file ijms-21-09374-s001.pdf]

Supplementary Material

Supplementary Table S1. Sequences of primers used for RTPCR and gene expression_

Additional details are in reference.

Function	Gene	Description	Sequence (L and R)
The biological actions of nuclear receptors	*VDR*	Vitamin D_3 receptor	*CTTACCTGCCCCCTGCTC*<br>*AGGGTCAGGCAGGGAAGT*
	RORA	Retinoic acid-related orphan receptor-α	GTCAGCAGCTTCTACCTGGAC<br>GTGTTGTTCTGAGAGTGAAAGGCACG
	RORC	Retinoic acid-related orphan receptor-γ	CAGCGCTCCAACATCTTCT<br>CCACATCTCCCACATGGACT
Inflammation	*TLR4*	Toll-like receptor 4	CAG GTG GAA TTG TAT CGC CT<br>CGA GGC TTT TCC ATC CAA TA
	COX2	Cyclooxygenase-2	GAATGGGGTGATGAGCAGTT<br>CAGAAGGGCAGGATACAGC
	ICAM	Intracellular adhesion molecule	CCTTCCTCACCGTGTACTGG<br>AGCGTAGGGTAAGGTTCTTGC
	IL6	Interleukin 6	GAAGCTCTATCTCGCCTCCA<br>AGCAGGCAACACCAGGAG
	IL8	Interleukin 8	AGACAGCAGAGCACACAAGC<br>ATGGTTCCTTCCGGTGGT
	IL17	Interleukin 17	TGGGAAGACCTCATTGGTGT

Function	Gene	Description	Sequence (L and R)
			GGATTTCGTGGGATTGTGAT
	IL33	Interleukin 33	TGTCAACAGCAGTCTACTGTGGAGTGCT<br>GCAAAAGTAATGGATTGATCATTGTA
	IL1A	Interleukin 1 alpha	GGTTGAGTTTAAGCCAATCCA<br>TGCTGACCTAGGCTTGATGA
	IL1B	Interleukin 1 beta	CRGTCCTGCGTGTTGAAAGA<br>TTGGGTAATTTTTGGGATCTACA
	IL10	Interleukin 10	TGGGGGAGAACCTGAAGAC<br>CCTTGCTCTTGTTTTCACAGG
	CD14	Cluster of differentiation 14	GTTCGGAAGACTTATCGACCAT<br>ACAAGGTTCTGGCGTGGT
	NFkB P50 (NFKB)	nuclear factor kappa B p50	*ACCCTGACCTTGCCTATTTG*<br>*AGCTCTTTTTCCCGATCTCC*
	NFkB P65 (RELA)	nuclear factor kappa B p65 (RELA)	*CGGGATGGCTTCTATGAGG*<br>*CTCCAGGTCCCGCTTCTT*
	IkBα (IKBA)	Inhibitory-kappa B-alpha	*GTCAAGGAGCTGCAGGAGAT*<br>*GATGGCCAAGTGCAGGAA*
	BCL2	B-cell lymphoma 2	AGTACCTGAACCGGCACCT<br>GGCCGTACAGTTCCACAAA
	BNIP	BCL2 adenovirus E1B interacting	CCGGGATGCAGGAGGAGAG<br>TTATAAATAGAAACCGAGGCTGGAAC

**Function**	**Gene**	**Description**	**Sequence (L and R)**
Differentiation	*IVL*	Involucrin	TGCCTCAGCCTTACTGTGAGT<br>TCATTTGCTCCTGATGGGTA
	LOR	Loricrin	GTGGGAGCGTCAAGTACTCC<br>AGAGTAGCCGCAGACAGAGC
	FLG	Filaggrin	GGCACTCATCATGCAGAGAA<br>ATGGTGTCCTGACCCTCTTG
	TGM1	Transglutaminase	TCTGTGGGTCCTGTCCCATCCATCCTGACC<br>CCCCAACGGCCCACATCGGAACGTGGCCCATCCATCATGC
	KRT1	Cytokeratin 1	GTTCCAGCGTGAGGTTTGTT TAAGGCTGGGACAAATCGAC
	KRT10	Cytokeratin 10	GGCTCTGGAAGAATCAAACTATGAGC<br>GGATGTTGGCATTATCAGTTGTTAGG
	KRT14	Cytokeratin 14	CTGTCTCCCGCACCAGCTTCACCTCC<br>CTCCACAAGCACCCGCAAGGCTGACC
	FGF23	Fibroblast growth factor 23	CAGCATGAGCGTCCTCAGAG GCCAGCATCCTCTGATCTGATC
House-keeping genes	*ACTB*	β-actin	CCAACCGCGAGAAGATGA<br>CCAGAGGCGTACAGGGATAG
	CYCB	cyclophilin B	TGTGGTGTTTGGCAAAGTTC<br>GTTTATCCCGGCTGTCTGTC
	GAPDH	Glyceraldehyde-3-Phosphate Dehydrogenase	AGCCACATCGCTCAGACAC<br>GCCCAATACGACCAAATCCC

Supplementary Table S2. List of antibody used for protein analysis

Function	Protein type	Catalog Number	Description	Dilution	Protein size (kDa)
Nuclear receptor	VDR	sc-13133 (Santa Cruz Biotechnology, Dallas, TX, USA)	mouse monoclonal antibody	*1:200*	*45*
	RORα	PA1-812 (Invitrogen, Oregon, USA)	rabbit polyclonal antibody	*1:200*	*56*
	RORγ	AFKJS-9 (Invitrogen, Oregon, USA)	mouse monoclonal antibody	*1:200*	*58*
Inflammation	NFkB p65	sc-8008 (Santa Cruz Biotechnology, Dallas, TX, USA)	mouse monoclonal antibody	1:1000	70
	IkB-α	sc-371 (Santa Cruz Biotechnology, Dallas, TX, USA)	rabbit polyclonal antibody	1:1000	37
Cell differentiation	IVL	GTX-14504 (Genetex, Irvine, CA, USA)	mouse monoclonal antibody	1:1000	68

Function	Protein type	Catalog Number	Description	Dilution	Protein size (kDa)
Loading control protein	β-actin-peroxidase	A3854 (Sigma-aldrich, St. Louis, MO, USA)	The whole and cytosolic loading control protein	1:5000	40
	Lamin A/C	sc6215 clone (N-18) (Santa Cruz Biotechnology, Dallas, TX, USA)	The nuclear loading control protein	1:2000	75
Secondary HRP antibody	Mouse HRP	A28177 (Thermo Fisher Scientific, Waltham, MA, USA)	goat anti-mouse IgG superclonal™-HRP	1:3000	-
	Rabbit HRP	ab6721 (Abcam, Cambridge, MA, USA)	goat anti-rabbit IgG-HRP	1:3000	-
Secondary fluorescence antibody	Alexa-Fluor 488	Alexa-Fluor 488 dye (Invitrogen Molecular Probes, Eugene, Oregon, USA)	Green fluorescence dye	1:300	-

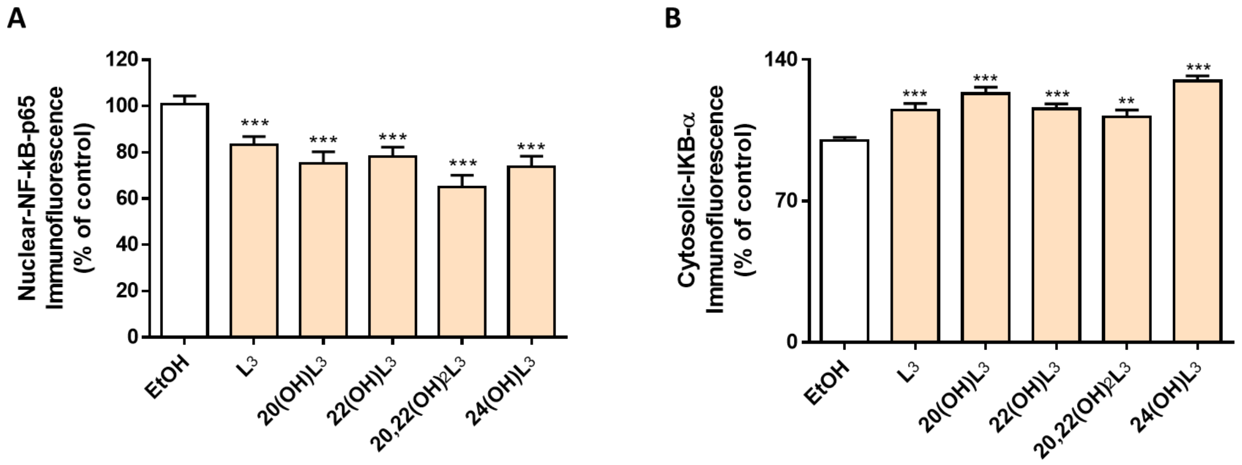

Fig. S1 Hydroxylumisterols display anti-inflammatory effects on UVB-irradiated keratinocytes by reducing nuclear NFκB p65 levels and increasing the levels of cytosolic-IκBα

Keratinocytes were treated with 100 nM hydroxylumisterols (shown as light orange bars in the chart) or ethanol (solvent control, shown as a white bar) for 24 h. Fluorescent microscopy of cells stained with (a) NFκB p65 and (b) cytosolic-IκBα antibodies was carried out using the Cytation™ 5 cell imaging, n ≥ 100 cells for each condition. Scale bar = 100 µm. Data are presented as the immunofluorescent staining of nuclear levels for NFκB p65 and the cytosolic-IκBα (% of control, mean ± S.D.). The statistical significance of differences was evaluated by the one-way ANOVA, ** $P < 0.01$ and *** $P < 0.001$, for all conditions relative to the untreated ethanol control.

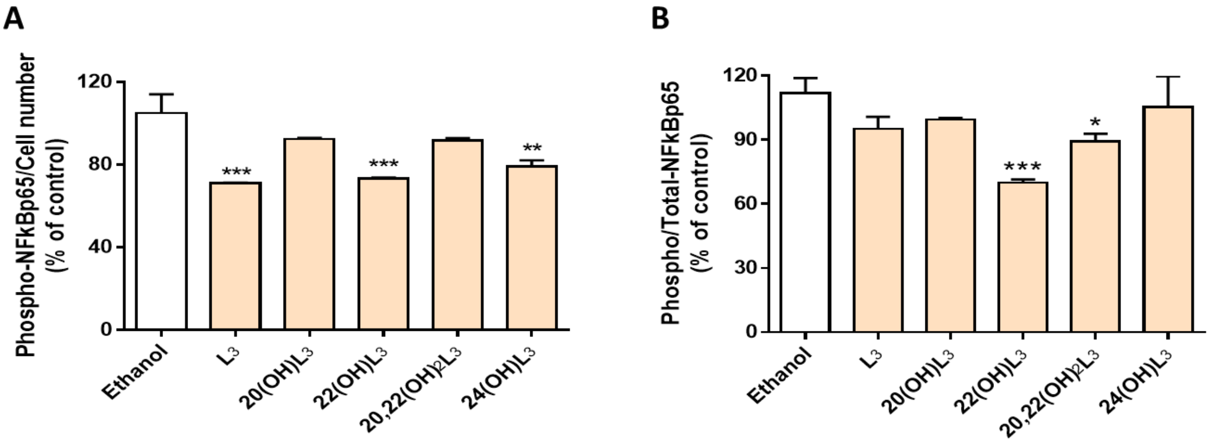

Fig. S2 Some of the hydroxylumisterols inhibit the phosphorylation of NFkB p65 in non-irradiated cells

Keratinocytes were treated with 100 nM hydroxylumisterols (light orange bars in the chart) or ethanol (solvent control, shown as a white bar) for 24 h. The phosphorylation of NFkB p65 and the total levels of NFkB p65 were determined by ELISA. (A) The levels of phospho-NFkB p65 normalized to cell number and (B) the phospho-NFkB p65 levels normalized relative to the total level of NFkB p65. The statistical significance of differences was evaluated by the one-way ANOVA, * $P < 0.05$ and ** $P < 0.01$, for all conditions relative to the untreated ethanol control, n=3.

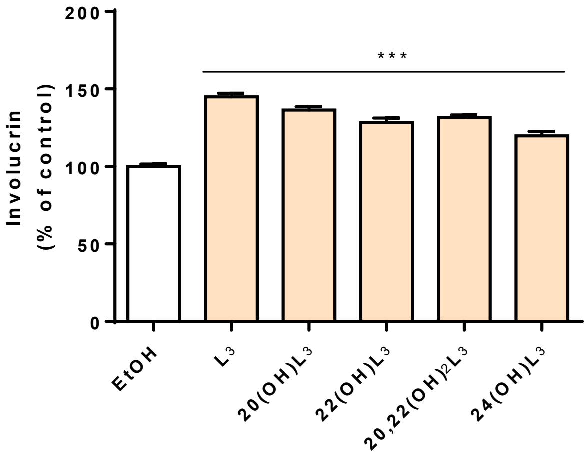

Fig. S3 Hydroxylumisterols promote keratinocyte differentiation in non-irradiated keratinocytes, as shown by increased levels of involucrin

Keratinocytes were treated with 100 nM hydroxylumisterols (light orange bars in the chart) or ethanol (solvent control, shown as a white bar) for 24 h. Fluorescent microscopy of cells stained with IVL was carried out using the Cytation™ 5 cell imaging, n ≥ 100 cells for each condition. Scale bar = 100 µm. Data are presented as the % of control for IVL (mean ± S.D.). The statistical significance of differences was evaluated by the one-way ANOVA, *** $P < 0.001$, for all conditions relative to the untreated ethanol control.

Reference:

1. Aoki, R.; Aoki-Yoshida, A.; Suzuki, C.; Takayama, Y. Protective Effect of Indole-3-Pyruvate against Ultraviolet B-Induced Damage to Cultured HaCaT Keratinocytes and the Skin of Hairless Mice. *PLoS ONE* **2014**, *9*, e96804, doi:10.1371/journal.pone.0096804.
2. Janjetovic, Z.; Jarrett, S.G.; Lee, E.F.; Duprey, C.; Reiter, R.J.; Slominski, A.T. Melatonin and its metabolites protect human melanocytes against UVB-induced damage: Involvement of NRF2-mediated pathways. *Sci. Rep.* **2017**, *7*, 1–13, doi:10.1038/s41598-017-01305-2.
3. Slominski, A.T.; Kim, T.-K.; Brożyna, A.; Janjetovic, Z.; Brooks, D.; Schwab, L.; Skobowiat, C.; Jóźwicki, W.; Seagroves, T. The role of melanogenesis in regulation of melanoma behavior: Melanogenesis leads to stimulation of HIF-1α expression and HIF-dependent attendant pathways. *Arch. Biochem. Biophys.* **2014**, *563*, 79–93, doi:10.1016/j.abb.2014.06.030.
4. Slominski, A.T.; Zmijewski, M.A.; Zbytek, B.; Brozyna, A.A.; Granese, J.; Pisarchik, A.; Szczesniewski, A.; Tobin, D.J. Regulated Proenkephalin Expression in Human Skin and Cultured Skin Cells. *J. Investig. Dermatol.* **2011**, *131*, 613–622, doi:10.1038/jid.2010.376.
